# Practice of Medicine

**Published:** 1860-09

**Authors:** 


### PRACTICE OF MEDICINE.

On the Rational Treatment of Delirium Tremens. By *Professor Dunglison*, of Philadelphia. In a letter to *Professor Laycock*.—[In the *Edinburgh Medical Journal* for October, 1858, I gave a series of cases of delirium tremens, all treated successfully without opium or alcoholic stimuli. I showed, too, that the delirium and sleeplessness indicate comparatively harmless conditions of the nervous system; that they are usually symptoms of some disease occurring in persons of drunken habits; that they usually cease within a given time, spontaneously; and that the proper method of cure is to treat the general or particular morbid state, whatever that may be, with which the delirium is associated. I further showed that opium and alcoholic stimuli, as generally recommended for this disease, were not only useless, but dangerous drugs. The paper has attracted considerable attention both at home and abroad, and will, I trust, have a beneficial effect in the management of this class of cases. The subjoined letter, addressed to me by Professor Dunglison, seems so well calculated to advance this object, that I have ventured to publish it. And I feel the more warranted in doing this, because it is simply an act of justice to Professor Dunglison, inasmuch as it proves that he was among the earliest advocates of a more rational method of treatment of delirium tremens.—T. L.]

PHILADELPHIA, *February* 21, 1860.

DEAR SIR:—Some time ago I had contemplated expressing to you the satisfaction I felt in perusing the views you entertained on the subject of the treatment of delirium tremens, of which I had, at one time, an opportunity of seeing much in the wards of the Philadelphia Hospital attached to the extensive Almshouse of this city, and containing upwards of 2000 inhabitants; but circumstances withdrew my attention from the subject, until it was revived by an article in a late number of the *British and Foreign Medico-Chirurgical Review*, and by Dr. Inman's recent work, entitled *Foundation for a New Theory and Practice of Medicine.*

It has been not a little gratifying to me to find that, without having seen what I have written on the subject, you should have arrived at results so nearly corresponding with those of my own observation. In the *American Medical Intelligencer* for May, 1842, of which I was editor, I inserted the following article, "by the Editor."

*On the Eclectic Treatment of Delirium Tremens.*—In a recent work, *Practice of Medicine*, vol. ii. p. 346, Philadelphia, 1842, we have stated that the course pursued by us in the treatment of delirium tremens has been entirely eclectic, in many cases expectant, and that the results have been such as to satisfy us. Under the view which we entertain of the nature of the affection—that the irregularity of nervous action is usually induced by the withdrawal of an accustomed stimulus, and that the recuperative powers are generally entirely sufficient to bring about the necessary equalization—we have treated the mass of the cases which have fallen under our care without either excitants proper, or opiates. In the first instance, an emetic is given at times, if the patient is seen while laboring under the effects of a debauch, or any particular reason exists for its administration; and afterwards, a state of tranquillity in the chamber is enjoined—the intrusion of too much light and noise being prevented; and, when the stomach will retain it, gently nutritious and easily digestible diet is prescribed, the bowels being kept open by gentle cathartics;—and this has comprised the essential part of our treatment. In time the hallucinations have disappeared, sleep has returned, and entire restoration supervened. The preceding remarks are a proper prelude to the statistical account of the Women's Lunatic Asylum, at the Philadelphia Hospital, for the years 1840 and 1841, which is under our charge during the six months commencing on the first of November and ending on the first of May, and under that of Dr. Pennock for the other half of the year. It may be proper to add, that since November 1, 1841, to the present time (May 1, 1842), not a drop of alcoholic liquor has been used in the treatment of delirium tremens in the Women's Asylum, although some severe cases in the third stage have occurred, which, notwithstanding, terminated most satisfactorily.

*Patients admitted into the Women's Lunatic Asylum of the Philadelphia Hospital.*

YEAR 1840.

| | Cases admitted. | Cured. | Died. |
|---|---|---|---|
| Intoxication | 25 | 25 | ... |
| Delirium Tremens, 1st stage | 34 | 34 | ... |
| " " 2d stage | 10 | 10 | ... |
| " " 3d stage | 4 | 3 | 1 |

The fatal case was not seen by us. The patient died on the morning after her admission into the hospital, and had been treated in the city for nearly a week previously.

YEAR 1841.

| | Cases admitted. | Cured. | Died. |
|---|---|---|---|
| Intoxication | 19 | 19 | ... |
| Delirium Tremens, 1st stage | 21 | 21 | ... |
| " " 2d stage | 9 | 9 | ... |
| " " 3d stage | 6 | 6 | ... |

In the third edition of my *Practice of Medicine*, (Philadelphia, 1848,) I state further: "A more recent authentic abstract of the number of patients admitted into the same asylum, from the 1st of November, 1844, to the 4th of February, 1845, exhibits that thirty-two cases were received, eighteen of which are classed as intoxication. Of these not one died. The treatment here again was eclectic, often expectant, and not a drop of alcohol was given."

The results of the above plan of treatment were referred to some time ago, in an interesting pamphlet on *Rational Medicine*, by Professor Worthington Hooker, of New Haven; but as I had doubts whether either the *Medical Intelligencer* or my *Practice of Medicine* is in the libraries of Edinburgh, I have copied from them what bears on the rational view which you have embraced of treating delirium tremens. As I have remarked in the latter work: "It has, in the first place, restored the individual to health, not, perhaps, as rapidly as either brandy or opium, but more permanently. The term 'restoration to health' is hardly, indeed, applicable to the change effected by the former remedy. The patient is merely placed in the condition in which he was before the stimulus was withdrawn; and as he was 'restored' by the brandy, he is apt, as before remarked, to regard it as indispensable to his healthy condition. In the 'total abstinence' plan, however, the habit of drinking is broken in upon; and even if it should require a short time longer to restore the individual, there is the consolatory reflection, that delay is not useless, and every day's privation of the wonted stimulus diminishes the feeling of necessity, and the desire for it. One evidence of the good effect of the course is, that they who are dismissed cured rarely or never return to the wards. This is an observation that has been made at the Philadelphia Hospital; and as it concerns paupers, it is probable that the cures are real and permanent, for, were it otherwise, they would, in subsequent attacks, be compelled, in their destitution, to seek the wards of the same excellent charity."

Pardon me, I pray you, for this long detail, which I hope may not be without interest to you, and believe me, with great respect, yours truly,

ROBLEY DUNGLISON.

